# Clinical Outcomes of Monoclonal Gammopathy of Renal Significance Without Detectable Clones

**DOI:** 10.1016/j.ekir.2023.09.022

**Published:** 2023-09-22

**Authors:** Maho Terashita, Umut Selamet, Shonali Midha, Omar Nadeem, Jacob Laubach, Helmut G. Rennke, Naoka Murakami

**Affiliations:** 1 Renal Division, Brigham and Women’s Hospital, Boston, Massachusetts, USA; 2 Harvard Medical School, Boston, Massachusetts, USA; 3 Division of Nephrology and Hypertension, St. Marianna University School of Medicine, Kanagawa, Japan; 4 Department of Medical Oncology, Dana Farber Cancer Institute, Boston, Massachusetts, USA; 5 Jerome Lipper Multiple Myeloma Center, Dana Farber Cancer Institute, Boston, Massachusetts, USA; 6 Pathology Department, Brigham and Women’s Hospital. Boston, Massachusetts, USA

**Keywords:** kidney biopsy, monoclonal gammopathy with renal significance, monoclonal immunoglobulin deposition disease, plasma cell dyscrasia, proteinuria, onconephrology

## Abstract

**Introduction:**

Monoclonal gammopathy of renal significance (MGRS) is characterized by monoclonal immunoglobulin deposition in kidneys. However, monoclonal immunoglobulin and responsible clone(s) are not always detectable. Treatment response and kidney outcome of MGRS without detectable clones remain unclear.

**Methods:**

In this single-center, retrospective cohort study, we identified MGRS without detectable clones from our biopsy repository between 2010 and 2022. We investigated the correlations between treatment regimens and kidney outcomes defined by proteinuria and estimated glomerular filtration rate (eGFR), and the impact of repeat kidney biopsy.

**Results:**

Our study cohort included 29 cases (27 native kidney and 2 transplant allograft biopsies) of MGRS without detectable clones. At diagnosis, median serum creatinine was 1.8 mg/dl (interquartile range [IQR] 1.3–2.7), with proteinuria 4.6 g/gCr (IQR 2.3–7.9). Treatment regimens were variable: 6 (21%) received conservative therapy, 13 (45%) received plasma cell clone-directed therapy, 8 (28%) received lymphocytic clone-directed therapy, and 2 (7%) received nonclone-directed immunosuppressive therapy. Of 24 patients with proteinuria >0.5 g/gCr at diagnosis, 9 (38%) and 6 (25%) achieved complete response (CR) and partial response (PR), respectively. If interstitial fibrosis and tubular atrophy (IFTA) was >50% at the initial biopsy, less proportion of patients achieved CR. Six of 7 repeat biopsies showed progression of chronic changes (e.g., IFTA) but provided limited information on treatment response.

**Conclusion:**

Treatment regimens and outcomes of MGRS without detectable clones were extremely variable. Repeat biopsy provided limited information to assess disease activity or the need for additional treatment. More sensitive tools are needed to detect clones and to assess treatment response.

## Graphical abstract

**Figure:**
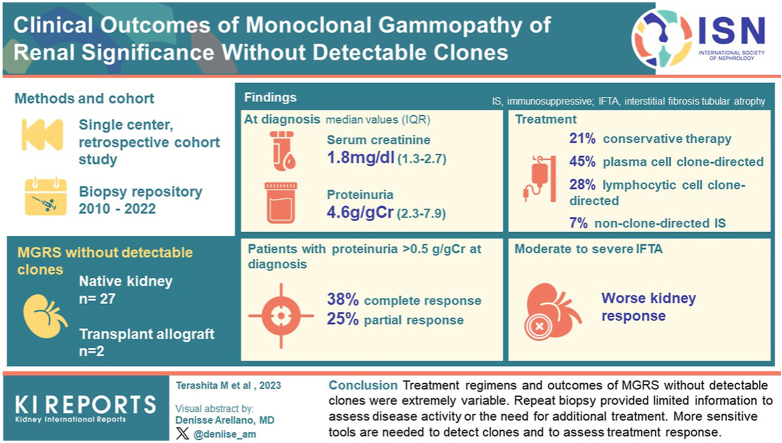

See Commentary on Page 2511

MGRS is a group of kidney diseases characterized by deposition of monoclonal proteins (immunoglobulin, light or heavy chains) in the kidney, where hematological diagnosis does not meet the current criteria of plasma cell dyscrasia or lymphoma, such as multiple myeloma, amyloidosis, or lymphoma.^1^ Identification of responsible clone(s) is critical to determine the treatment strategies, because the mainstay of treatment involves the directed therapy against plasma cell or B cell clone(s) that produce the monoclonal protein.^2^ However, in some cases of MGRS, monoclonal protein is not detectable in serum, urine, or bone marrow, and this sets clinical challenges on whether and how to treat the condition due to lack of biochemical and cytologic markers to follow clinical response.^1^^,^^3^

The clone identification rate depends on MGRS subtypes. Especially in those with histopathological patten of proliferative glomerulonephritis with monoclonal immunoglobulin deposits (PGNMID), the clone identification rate is reportedly as low as 30%.^1^ Although some retrospective cohort studies reported a moderate response rate of MGRS against lymphocyte clone-directed therapies,^2^^,^^4^ kidney outcomes of MGRS without detectable clones in particular, are not entirely clear. In this study, we investigated kidney outcomes of MGRS without detectable clones, using a single-center, retrospective case series of 29 patients.

## Methods

### Inclusion Criteria

We identified kidney biopsy cases performed between January 1, 2010, and December 31, 2022, that included the term “monoclonal immunoglobulin deposition” or “monoclonal paraprotein deposition” in the pathology report, using the kidney biopsy pathology database at Brigham and Women’s Hospital. According to the standard of practice, all kidney biopsy samples were evaluated for light, immunofluorescence, and electron microscopy. The description of monoclonal immunoglobulin deposition on the pathology reports was used for the following findings: (i) deposition of monotypic immunoglobulins and/or light chains in the glomeruli and/or tubular basement membranes by immunofluorescence and (ii) electron-dense deposits in mesangial, subendothelial, or subepithelial locations and/or tubular basement membranes.

We reviewed demographic information, clinical and laboratory findings, including the presence of serum and urine monoclonal protein, bone marrow biopsy and flow cytometry. Urine protein level was measured as either 24-hour urine collection or random urine protein-creatinine ratio, whichever was available. The hematologic evaluation included serum protein electrophoresis (SPEP) with immunofixation (IFX), urine protein electrophoresis, serum free light chain (FLC) assay, and bone marrow biopsy. Normal values for the FLC ratio were 0.26 to 1.65 for eGFR ≥60 ml/min per 1.73 m^2^ and 0.37 to 3.1 for eGFR <60 ml/min per 1.73 m^2^.^5^^,^^6^ eGFR was calculated by the Chronic Kidney Disease-Epidemiology Collaboration equation.^7^ If eGFR was unavailable on the same day that FLC was measured, the value from the closest day was used. Patients with specific hematological diagnosis (e.g., multiple myeloma, amyloidosis, or other lymphoproliferative diseases) were excluded from the analysis. We included the cases of MGRS with undetectable clones and monoclonal protein when they met all 4 criteria: (i) negative SPEP with IFX, (ii) normal serum FLC ratio adjusted for kidney function, (iii) no clones identified on bone marrow examination, and (iv) criteria for specific hematologic diagnosis, such as amyloidosis, smoldering or overt multiple myeloma, or lymphoma not met.

### Evaluation of Kidney Outcomes

The kidney response to treatment is defined by the improvement in proteinuria.^2^ CR is defined when proteinuria improved to <0.5 g/gCr on urine protein-to-creatinine ratio or in 24-hour urine collection with stable kidney function (deterioration in eGFR of less than 20%). PR is defined as >50% reduction of proteinuria compared to the time of the first kidney biopsy and less than 3 g/gCr on urine protein-to-creatinine ratio or 24-hour urine collection with stable kidney function. The best kidney response during follow-up period was reported unless otherwise documented. The time from diagnosis to the kidney response (PR or CR) was also recorded. If proteinuria at diagnosis was less than 0.5 g/gCr on urine protein-to-creatinine ratio or 24-hour urine collection, it was defined as non-available. If none of the above definitions were applied, the patient was described as having no response. End-stage kidney disease (ESKD) was defined as a requirement of renal replacement therapy.

## Results

### Demographic and Clinical Characteristics

We identified 493 kidney biopsy cases that included “monoclonal immunoglobulin deposition” or “monoclonal paraprotein deposition” in the pathology report, of which 223 had monoclonal immunoglobulin deposition as a primary diagnosis and 91 cases had a longitudinal clinical and laboratory follow-up data at Mass General Brigham and Dana Farber Cancer Institute. Twenty-nine cases of MGRS, 27 in native kidneys and 2 in transplanted kidneys, without detectable monoclonal proteins and clones were included in this study. Seven patients underwent multiple kidney biopsies. Demographic and clinical presenting characteristics are listed in Table 1. The median age was 58 (IQR, 47–66) years and 52% were male. The median serum creatinine at diagnosis was 1.8 mg/dl (IQR 1.3–2.7 mg/dl). Of note, only 1 patient had severe acute kidney injury and initiated hemodialysis immediately after biopsy. The median proteinuria was 4.6 g/gCr on urine protein-to-creatinine ratio or in 24-hour urine collection (IQR 2.3–7.9 g/gCr). Two patients met the definition of nephrotic syndrome at the time of diagnosis. All patients underwent SPEP with IFX, and serum FLC evaluation, which confirmed the absence of monoclonal immunoglobulin in blood. Majority (86%, 25 of 29) underwent bone marrow biopsy as a part of work-up. Some patients had additional work-up to look for clones: 13 patients underwent positron emission tomography–computed tomography, none of whom had fluorodeoxyglucose-avid lesions. Four patients underwent peripheral blood flow cytometry. One had a small number of B cells with monotypic cytoplasmic lambda immunoglobulin light chain expression in the peripheral blood, assumed as monoclonal B-cell lymphocytosis.

**Table1:** Clinical characteristics

Total 29 (native kidneys 27, transplanted kidneys 2)	
Median age (IQR), yrs	58 (47–66)
Male (%)	15 (52)
Diagnostic year	
2010–2013	5
2014–2016	8
2017–2019	6
2020–2022	10
Cre at presentation,median (IQR), mg/dl	1.8(1.3, 2.7)
Proteinuria at presentation,median (IQR), g/24h or g/gCr	4.6(2.3, 7.9)
Plasma cells in bone marrow	
N/A	6
none	2
<5%	16
5–10%	4
>10% (polyclonal)	1
Histopathological pattern	
MPGN	18
MSGN	5
MN	3
PGN	3
Monoclonal deposition on the first kidney biopsy	
IgG/KAPPA	2
IgG/LAMBDA	1
IgG1(heavy chain only)	1
IgG1/KAPPA	5
IgG1/LAMBDA	3
IgG2/KAPPA	1
IgG3/KAPPA	7
IgG3/LAMBDA	6
IgA/KAPPA	3
Interstitial fibrosis and tubular atrophy (IFTA)	
<25%	17
25–50%	6
>50%	6
Treatment(Initial regimen)	
Conservative therapy	8
Plasma cell clone-directed therapy	
CyBorD	4
DaraCyBorD	1
Lenalidomide+PSL	1
Lymphocytic clone-directed therapy	
R	5
R+PSL	1
R+PSL+CY	1
Nonclone-directed therapy	
Steroid monotherapy	5
PSL+CY	2
PSL+MMF	1
Adverse events	
Plasma cell clone-directed therapy	
Neutropenia	3
Nausea	3
Fatigue	3
Local skin reaction	2
Insomnia	2
Severe hypertension	2
Rash	2
GI symptom	2
Fluid retention	2
Pneumonia	2
Blepharitis	1
Peripheral neuropathy	1
Light headache	1
Acute kidney injury	1
Arthralgia	1
Cellulitis	1
Lymphocytic clone-directed therapy	
Nausea	2
Flu-like illness	1
JCV infection	1
Neutropenia	1
Nonclone-directed therapy	
Esophagitis	1
Fatigue	1
Headache	1
Hyponatremia	1
Nausea	1
Neutropenia	1

BD, bortezomib and dexamethasone; Cre, creatinine; CY, cyclophosphamide; CyBorD, cyclophosphamide, bortezomib, and dexamethasone; DaraCyBorD, daratumumab, cyclophosphamide, bortezomib, and dexamethasone; GI, gastrointestinal; IFTA, interstitial fibrosis and tubular atrophy; IQR, interquartile range; JCV, John Cunningham virus; MMF, mycophenolate mofetil; MN, membranous pattern; MPGN, membranoproliferative glomerulonephritis; MSGN, mesangioproliferative glomerulonephritis; N/A, not available; PGN, proliferative glomerulonephritis; PSL, prednisone; R, rituximab.

The 3 cases with IgG/KAPPA or IgG/LAMBDA deposition did not have subclass determination because we did not have the reagents to determine the IgG subclasses at that time.

**Figure 1:**
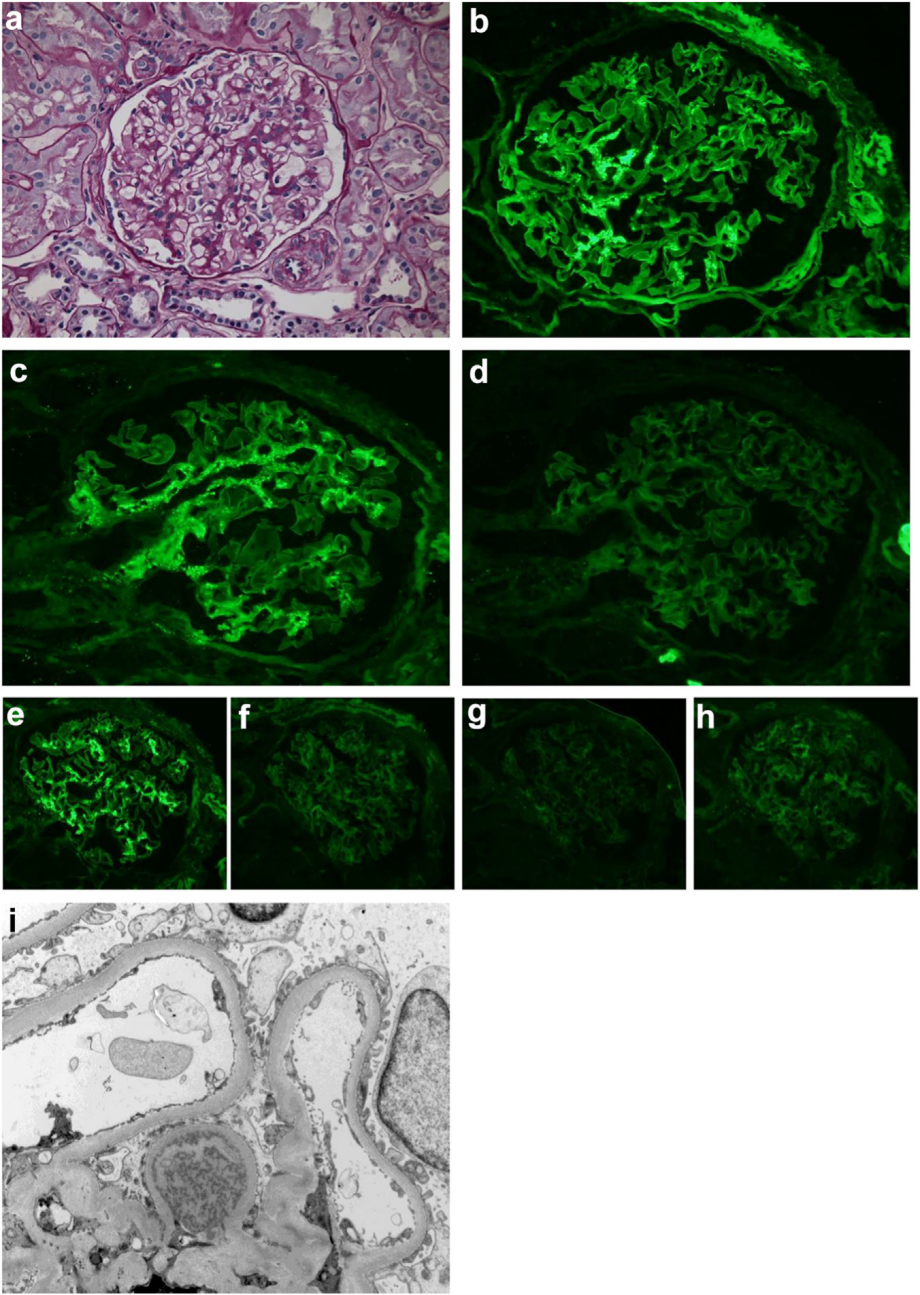
Monoclonal IgG1/kappa deposition disease with a mesangioproliferative pattern of injury. (a) There is mild mesangial expansion predominantly due to an increase in mesangial matrix (PAS). There is confluent granular deposition of IgG (b) in the mesangium (anti-IgG). The deposits are reactive for kappa (c) light chain and gamma1 heavy chain (e) only, and they do not react for lambda light chain (d), and gamma2 (f), gamma3 (g), and gamma4 (h) heavy chains. (i) Electron dense deposits are limited to the mesangium, and they reveal variable electron density that has resulted in a variegated appearance of the deposits. (Magnifications: a = ×175; b, c, and d = ×200; e, f, g, h = ×100; i = ×5700).

**Figure 2:**
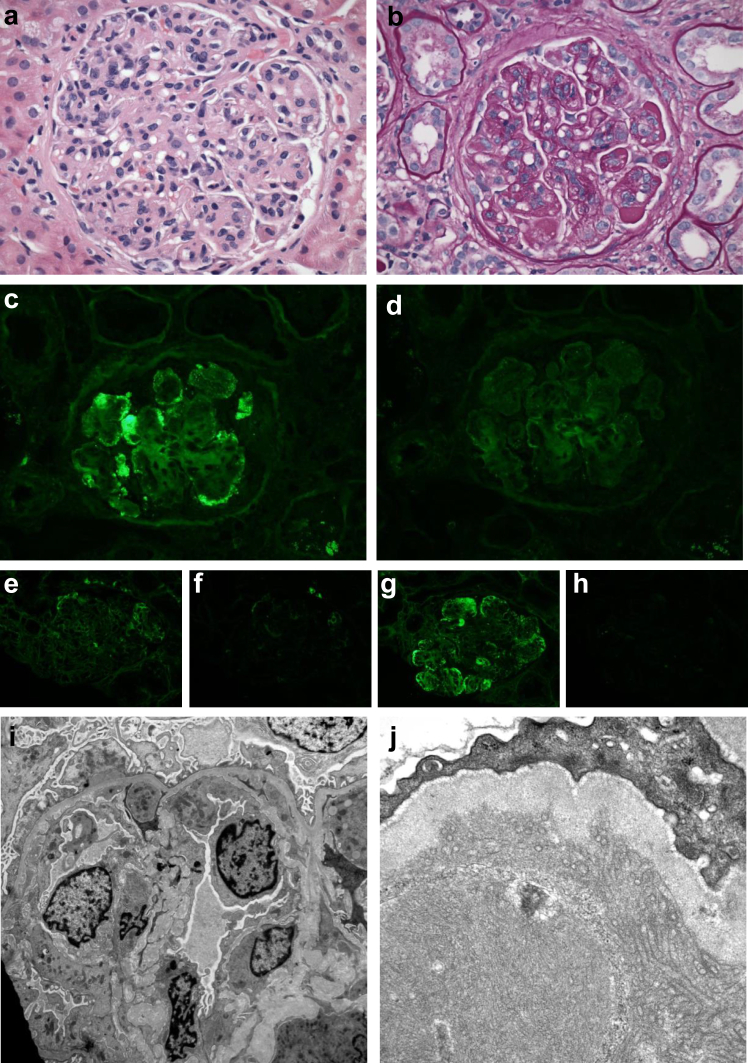
Monoclonal IgG3/kappa deposition disease with a diffuse proliferative and membranoproliferative pattern of injury. (a) There is global hypercellularity of the tuft with predominance of mononuclear inflammatory cells within the glomerular capillaries (HE). (b) The PAS stain highlights the thickened peripheral capillary walls, that show frequent duplication of the basement membranes (double contours). The immunofluorescence microscopy shows capillary wall deposits reactive for kappa (c) but not lambda (d) light chains. These deposits are restricted for gamma3 heavy chain (g), and are non-reactive for gamma1 (e), gamma2 (f), and gamma4 (g) heavy chains. (i) The electron microscopy reveals capillaries with swollen endothelium and infiltrating mononuclear inflammatory cells, double contours of the capillary walls, and subendothelial and isolated subepithelial deposits of low to medium electron density. (j) At higher magnification, the deposits reveal an organized substructure with microtubular arrays. (Magnifications: a, b, c, and d = ×200; e, f, g, and h =×100; i = ×4300; j = ×20,000).

**Figure 3:**
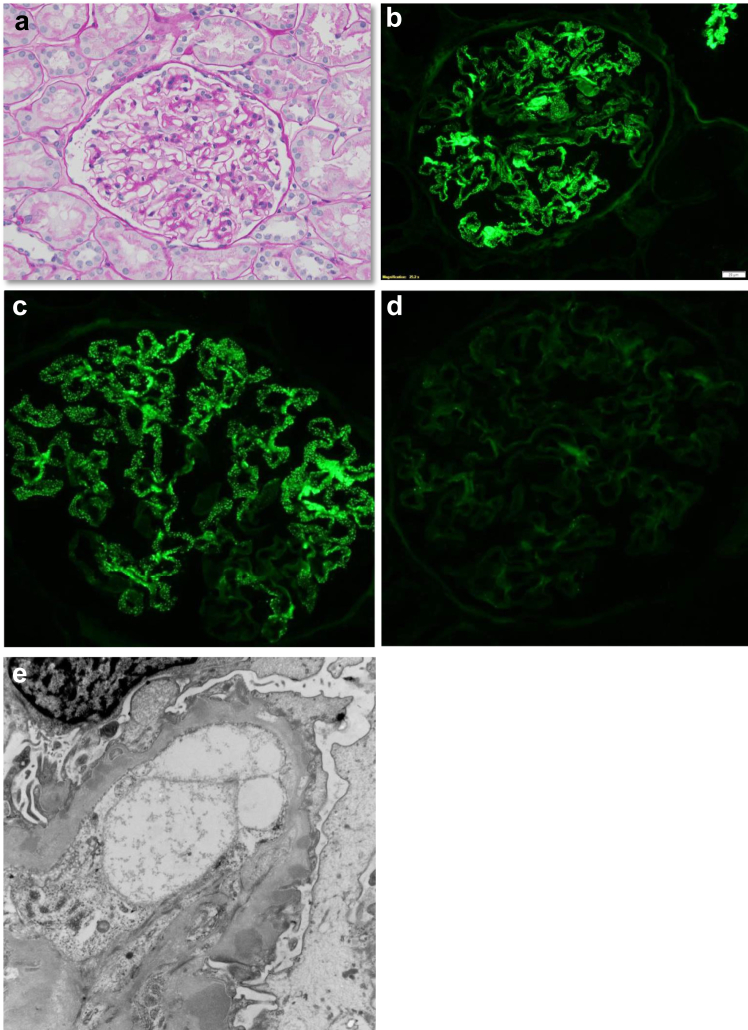
Monoclonal IgG1/kappa deposition disease with a membranous pattern of injury (a) The glomerulus shows normal cellularity and minimal thickening of the basement membrane son PAS stain. (b) There is fine granular deposition of IgG mostly along the peripheral glomerular capillary walls. The deposits are restricted for kappa light chains (c) and for gamma1 (not illustrated). The reactivity of the deposits for lambda light chain (d) is negative. The electron microscopy shows mostly subepithelial electron dense deposits and marked simplification and effacement of the glomerular visceral epithelial cell foot processes. (Magnifications: a and b = ×175; c and d = ×225; e = ×6900.

The histopathological patterns of injury in 29 biopsies were as follows: 18 cases showed a membranoproliferative pattern of injury, 5 cases had a mesangioproliferative glomerulonephritis, 3 cases revealed a membranous pattern, and 3 cases showed a proliferative glomerulonephritis (Table 1). Characteristic histopathological images are shown in Figure 1, Figure 2, Figure 3. The most frequent immunoglobulin deposited in the kidneys was IgG3/Kappa. All patients except 1 had both heavy and light chain depositions on immunofluorescence and were clinically diagnosed as PGNMID. One patient who had IgG1 deposition alone on glomeruli showed membranous pattern on light microscopy (heavy chain deposition disease). In our cohort, no patient had deposition in the tubular basement membrane on immunofluorescence and electron microscopy. IFTA were mild (less than 25%) in 17 cases, moderate (25%–50%) in 6 cases, and severe (>50%) in 6 cases (Table 1).

### Treatment Regimens

**Figure 4:**
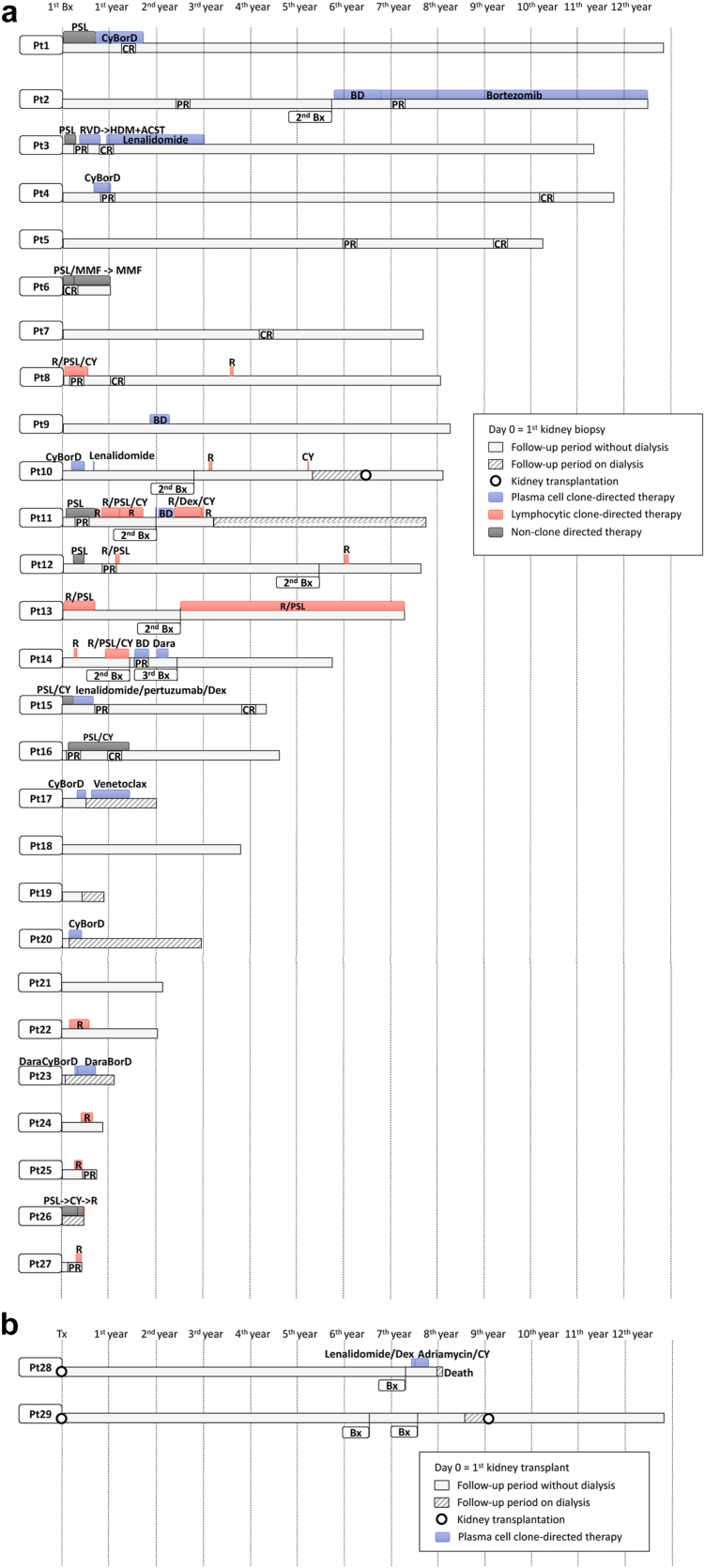
Patient clinical course, treatment history and response. (a) Patients with native kidneys. Day 0 (left side of the timeline) is the day of the first kidney biopsy. (b) Patients with transplanted kidneys. Day 0 (left side of the timeline) is the day of the first kidney transplantation. ASCT, autologous stem-cell transplantation; BD, bortezomib and dexamethasone; Bx, biopsy; CR, complete response; CY, cyclophosphamide; CyBorD, cyclophosphamide, bortezomib, and dexamethasone; Dara, daratumumab; DaraBorD, daratumumab, bortezomib and dexamethasone; DaraCyBorD, daratumumab, cyclophosphamide, bortezomib, and dexamethasone; Dex, dexamethasone; HDM, high dose melphalan; MMF, mycophenolate mofetil; PR, partial response; PSL, prednisone; Pt, patient; R, rituximab; RVD, lenalidomide, bortezomib, and dexamethasone.

The treatment regimens were divided into 4 categories. Conservative therapy category refers to the absence of any specific treatment for MGRS. This category includes conservative management with renin-angiotensin-aldosterone system inhibitors. Plasma cell clone-directed therapy category includes bortezomib, daratumumab, or their combination regimen of cyclophosphamide/ bortezomib/ dexamethasone. Lymphocytic clone-directed therapy category refers to rituximab-based regimens. Nonclone-directed therapy category includes glucocorticoids, oral cyclophosphamide, and mycophenolate mofetil. The first line treatment regimens are shown in Table 1. Treatment histories in detail of each patient with native kidneys are shown in Figure 4a, and those of patients with transplanted kidneys are shown in Figure 4b.

Eight (28%) patients started with conservative therapy. One was a transplant recipient under maintenance immunosuppression with tacrolimus and mycophenolate mofetil. Of the 7 nontransplant patients, 3 had spontaneous improvement in proteinuria and improved serum creatinine levels without specific treatment.

Six (21%) patients received plasma cell clone-directed therapy from the beginning. One was a posttransplant case. Cyclophosphamide/ bortezomib/ dexamethasone regimen was most frequently used in this group. One patient (patient #3) received high-dose melphalan and autologous stem cell transplantation. The patient presented with over 15 g/gCr of proteinuria and kidney biopsy revealed IgG/lambda monoclonal deposition with diffuse proliferative pattern of injury and was diagnosed as PGNMID. The patient was treated with a course of prednisone, which resulted in only slight improvement in proteinuria and lower extremity edema. The patient was then treated with lenalidomide, bortezomib, and dexamethasone. After 3 cycles, the proteinuria decreased to 1.3 g/gCr. The patient suffered from neuropathy due to bortezomib, and a decision was made to proceed with high-dose melphalan and autologous stem cell transplantation. The patient underwent a course of posttransplant maintenance therapy with lenalidomide for 3 years and achieved CR in proteinuria.

Seven (24%) patients were initially treated with lymphocytic clone-directed therapy. Five patients were treated with rituximab monotherapy; 1 was treated with prednisone and rituximab; and 1 was treated with prednisone, oral cyclophosphamide, and rituximab.

Eight (28%) patients started with nonclone directed therapy. Five were treated with prednisone monotherapy; 2 were treated with prednisone and oral cyclophosphamide. All patients whose first-line treatment was glucocorticoid monotherapy received second-line therapy.

**Figure 5:**
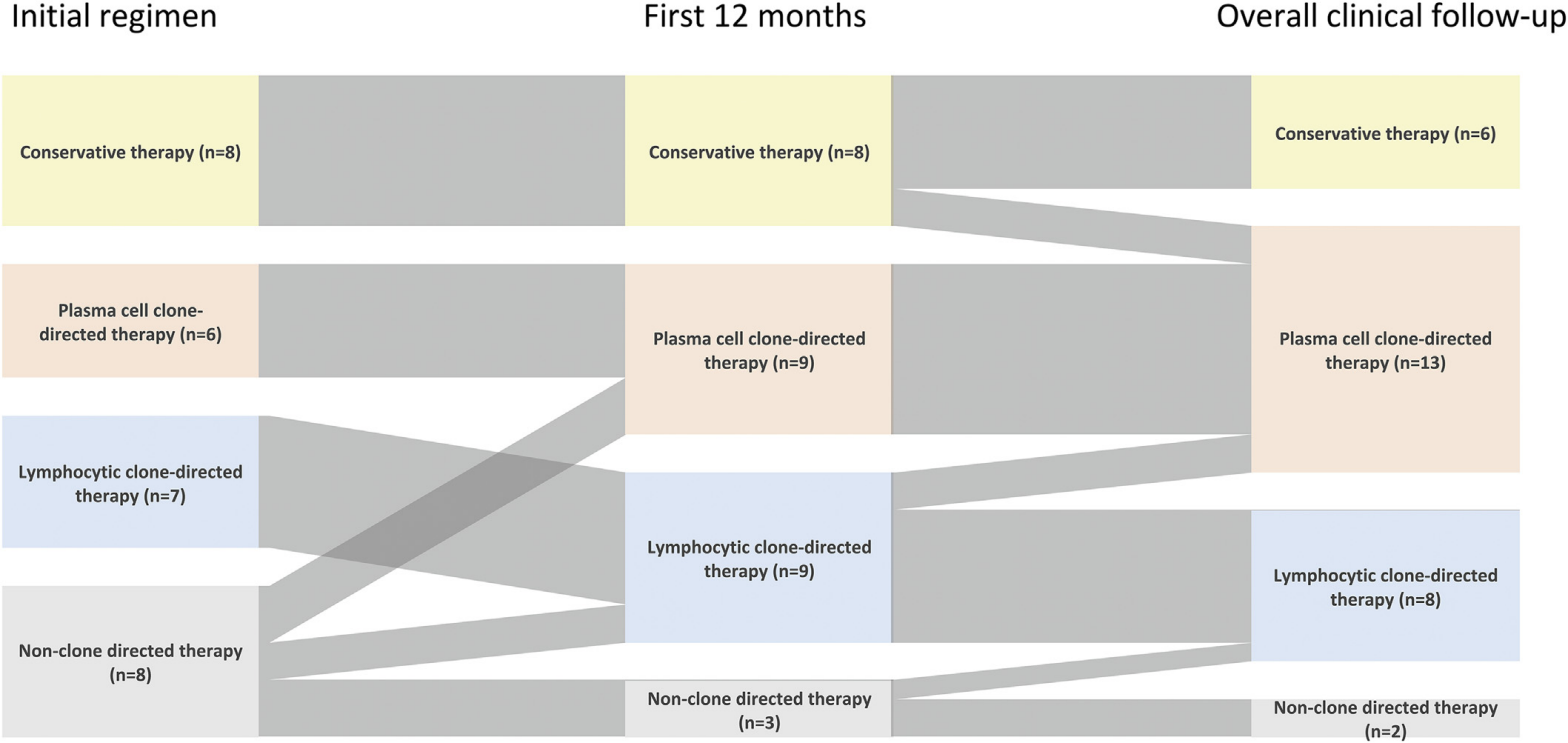
Changes in treatment regimens. Sankey diagram shows the changes in treatment regimens at the initial intervention (left), at 12-month after biopsy (middle), and at the overall clinical follow-up period (right). For the 12-month and the overall clinical follow-up timepoints, the therapy categories are “upgraded” to the most intensive regimen they received. For example, if the patients received plasma cell clone-targeted therapy at any timepoint, the patients were categorized as a “plasm cell clone-targeted therapy” group.

Some patients got transitioned to another treatment group during the treatment course. In the conservative therapy group, 2 patients were switched to plasma cell clone-directed therapy. In lymphocytic clone-directed therapy group, 1 patient transitioned to plasma cell clone-directed therapy. In nonclone-directed therapy group, 4 patients eventually had plasma cell clone-directed therapy; and 2 had rituximab. The changes in treatment regimens are summarized in Figure 5.

The adverse events in each treatment group are also shown in Table 1. Three patients experienced severe adverse events that required treatment discontinuation: severe hypertension by lenalidomide, diffuse rash by bortezomib, headache and hyponatremia by cyclophosphamide.

### Kidney Outcomes

**Figure 6:**
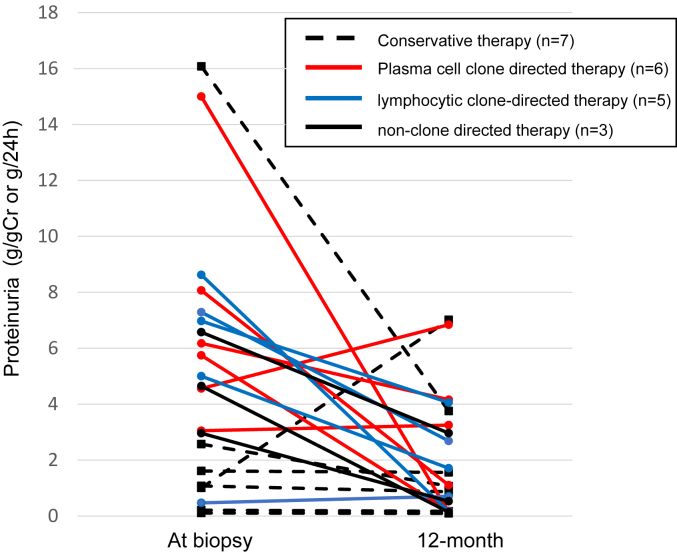
Change in proteinuria after 12 months from diagnosis. Twenty-one patients whose urine protein quantification was available at 12 months after kidney biopsy were included in the analysis. Patients with their follow-up less than 12 months (*n* = 5) and who were on dialysis at 12-month timepoint (*n* = 3) were excluded from the analysis. For treatment groups, those who received at least 1 plasma cell clone-directed therapy in the first 12 months were classified as the plasma cell clone-directed therapy group.

We first evaluated the change in proteinuria at 12-month after diagnosis for 21 patients whose proteinuria was available at 12 months after the biopsy (Figure 6). Those who received at least 1 plasma cell clone-directed therapy in the first 12 months were classified as the plasma cell clone-directed therapy group. Patients who received rituximab without plasma cell clone-directed therapy in the first 12 months were classified as a lymphocytic clone-directed therapy group. Proteinuria decreased or remained stable in most patients regardless of the types of treatments.

**Figure 7:**
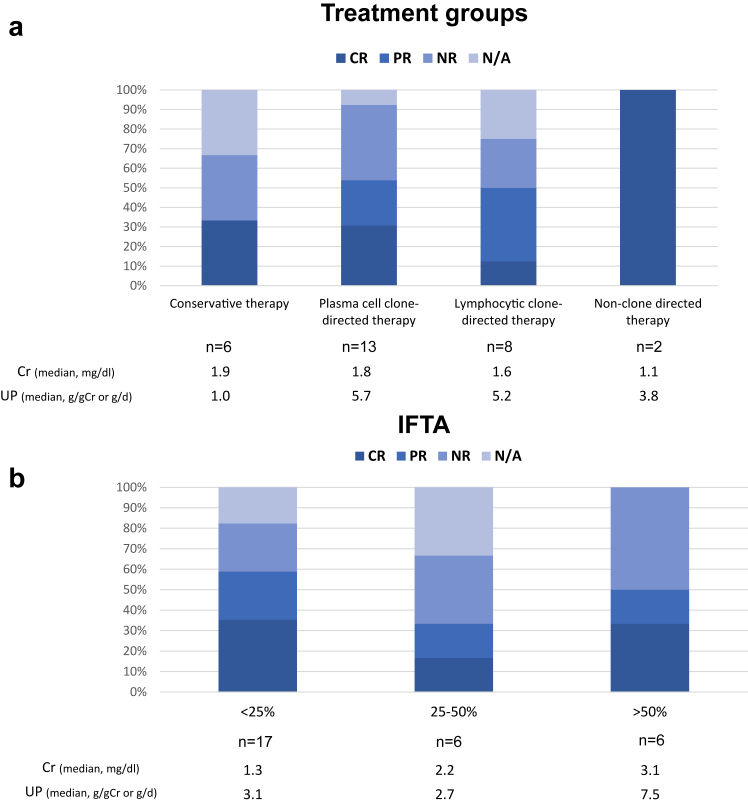
Treatment response. Kidney treatment response in patients with native kidneys who have proteinuria over 0.5 g/gCr at diagnosis. (a) categorized by therapy regimen. (b) categorized by IFTA at diagnosis. Those who received at least 1 plasma cell clone-directed therapy during all follow up were classified as the plasma cell clone-directed therapy group. Patients whose proteinuria at diagnosis was less than 0.5 g/gCr on urine protein-to-creatinine ratio or 24-hour urine collection were categorized as not-applicable (N/A). CR, complete response; IFTA, interstitial fibrosis and tubular atrophy; N/A, not applicable; NR, no response; PR, partial response.

We next evaluated the best kidney outcomes by proteinuria, according to the treatment groups (Figure 7a). Those who received at least 1 plasma cell clone-directed therapy during the entire follow-up period were reclassified as the plasma cell clone-directed therapy group. The median duration of follow-up was 55 months (range 5–154 months). In the conservative therapy group, 2 patients achieved CR at 4.4 and 9.4 years. In the plasma cell clone-directed therapy group, 3 patients achieved PR, and 4 achieved CR. In lymphocytic clone-directed therapy group, 3 patients achieved PR, and 1 achieved CR. We also analyzed the kidney outcomes by the severity of IFTA on initial kidney biopsies (Figure 7b). Six of 17 patients with mild IFTA (<25%) achieved CR, and 4 achieved PR. Of the 6 patients who had had moderate IFTA (25%–50%), 1 achieved CR, and 1 achieved PR. Two patients reached CR and 1 reached PR among the 5 patients who showed severe IFTA (>50%). All the follow-up courses with detailed treatment regimens are presented in Figure 4a.

Of the 21 nontransplant patients whose eGFR was less than 50 ml/min per 1.73 m^2^ at the time of initial kidney biopsy, only 3 had recovered to an eGFR of 60 ml/min per 1.73 m^2^ or higher, and all of these patients were in the lowest IFTA group. Seven of the 26 patients progressed to ESKD despite undergoing some form of treatment. The median time from diagnosis to ESKD was 4 months (IQR 0.5–21.5). One patient had already developed ESKD at diagnosis and did not recover.

### Kidney Outcomes in Patients With Transplanted Kidneys

The outcomes of patients with transplanted kidneys are shown in Figure 4b. Patient #28 was diagnosed with membranoproliferative glomerulonephritis with monoclonal immunoglobulin deposition on the native kidney biopsy. The patient had been treated with lenalidomide/dexamethasone before kidney transplantation. The patient developed histopathologic recurrence approximately 7 years after the kidney transplant and had Merkel cell carcinoma simultaneously. Lenalidomide and dexamethasone were started; however, the Merkel cell carcinoma eventually progressed and the patient died. The primary kidney disease of patient #29 was thought to be a congenital solitary kidney, and the patient had not undergone the native kidney biopsy. The patient had an allograft biopsy due to a slowly worsening creatinine 7 years after the transplantation, which revealed monoclonal IgG1 kappa deposition in glomeruli. The patient was kept under close surveillance without additional treatment nor change in immunosuppression regimen. After 12 months of conservative therapy, the patient underwent another graft biopsy, which showed spontaneous resolution of monoclonal deposition. Severe IFTA was characterized on the second biopsy. The patient developed ESKD and had a second kidney transplantation 8.6 years after transplantation. The patient has not shown any sign of MGRS since the second kidney transplant.

### Role of Repeat Kidney Biopsies

Finally, we assessed whether the repeat kidney biopsy would add clinical information for deciding the treatment choice and length. Seven patients in our cohort had more than 1 kidney biopsies (Table 2). The reasons for the repeat biopsies were worsening kidney function and/or overt proteinuria. A third kidney biopsy of patient #14 was performed to evaluate the activity of the disease, because serum creatinine and proteinuria remained unchanged despite treatment, and disease activity could not be evaluated with other markers. Only 1 patient with the transplanted kidney had monoclonal deposition disappear. In the other patients, there were no changes in glomerulonephritis patterns and monoclonal deposition. All patients had progression of IFTA and global sclerosis regardless of treatment, except for 1 patient. In 4 patients, the treatment regimen was changed to another group after the repeat biopsy. Patient #14 received several lines of treatment, but after the third kidney biopsy showed no change in findings, it was decided to discontinue treatment.

**Table 2:** Cases with repeat biopsies

No.	Time from 1^st^ biopsy (yr)	Age(yr)	Cre (mg/dl)	UP (g/gCr or 24h)	Reason for rebiopsy	IF	LM	GS (%)	IFTA (%)	FLC ratio	SPEP	Treatment
2	-	42	1.4	2.57	-	IgA/KAPPA	MSGN	25	10–20	0.80	No M-spike	Conservative therapy
2	5.71	48	2.47	2.19	Worsening Cre	IgA/KAPPA	MSGN	84	50–60	1.44	No M-spike	PC-directed
10	-	47	1.11	4.56	-	IgG3/KAPPA	MPGN	26	20–30	0.78	No M-spike	PC-directed
10	2.81	49	1.43	4.72	Persistent UP, worsening Cre	IgG3/KAPPA	MPGN	44	50–60	1.33	No M-spike	LC-directed
11	-	69	1.47	5.00	-	IgG3/KAPPA	MPGN, nodular	20	30	1.88	No M-spike	LC-directed
11	1.99	71	2.57	2.99	Worsening Cre	IgG/KAPPA	MPGN, nodular	39	50	2.58	Faint IgG Kappa	PC-directed
12	-	31	1.41	6.58	-	IgG2/KAPPA	PGN	13	10–20	1.57	No M-spike	LC-directed
12	5.48	37	1.52	3.95	Persistent UP	IgG2/KAPPA	PGN	25	20	N/A	N/A	LC-directed
13	-	60	1.28	0.47	-	IgG1/LAMBDA	PGN, crescentic	36	10–20	1.32	No M-spike	LC-directed
13	2.52	63	3.43	1.06	Worsening Cre & UP	IgG1/LAMBDA	PGN, crescentic	48	60–70	0.99	No M-spike	LC-directed
14	-	26	0.47	7.29	-	IgG1/KAPPA	MPGN	0	Minimal	0.88	No M-spike	LC-directed
14	1.44	27	0.45	3.76	Persistent UP	IgG1/KAPPA	MPGN	10	5	0.96	No M-spike	PC-directed
14	2.44	28	0.57	4.12	Determination of treatment effect	IgG1/KAPPA	MPGN	6	<5	1.33	spike in γ lesion	Conservative therapy
29	-	69	1.29	1.00	-	IgG1/KAPPA	MPGN	25	10–20	1.72	No M-spike	Conservative therapy
29	1.05	70	1.84	7.02	Worsening Cre & UP	NONE	collapsing	42	60	2.28	No M-spike	Conservative therapy

Cre, creatinine; FLC, free light chain; GS, global sclerosis; IF, immunofluorescence; IFTA, interstitial fibrosis and tubular atrophy; LC, lymphocytic clone; LM, light microscopy; PGN, proliferative glomerulonephritis; MPGN, membranoproliferative glomerulonephritis; MSGN, mesangioproliferative glomerulonephritis; PC, plasma cell clone; SPEP, serum protein electrophoresis; UP, urine protein; collapsing, collapsing glomerular lesions or collapsing GN; Y, year.

## Discussion

In this report of single-center case series, we describe the treatment regimens and clinical outcomes of MRGS without detectable clones seen at Brigham and Women’s Hospital or Dana Farber Cancer Institute between 2010 and 2022. The treatment practice patterns and kidney outcomes were highly variable.

Treatment approaches for MGRS without detectable clones vary from conservative therapy to nonclone directed immunosuppressive therapy generally used for glomerulonephritis, lymphocytic clone-directed therapy (e.g., rituximab), and plasma cell clone-directed therapy. In our cohort, we describe 8 patients with conservative therapy, 6 with plasma cell clone-directed therapy, 7 with lymphocytic clone-directed therapy, and 8 with nonclone-directed therapy as the first line treatment. In some situations, patients who did not respond well to the initial treatment were switched to plasma cell clone-directed therapy or lymphocytic clone-directed therapy from other therapy groups (Figure 5). We summarized published studies that evaluated the treatments and kidney outcomes of each MGRS case without detectable clones (Table 3).^2^^,^^8^, ^9^, ^10^ Relative to these studies, plasma cell clone-directed therapy was used more frequently in our cohort. Overall, our study redemonstrated variable practice patterns in MGRS without detectable clones.

**Table 3:** Summary of MGRS patients without detectable clones in the literature review

Study	Treatment	Cases	Outcome CR/PR/NR	ESKD
Guiard *et al.*^9^	Conservative therapy	3	0/0/3	3
	Plasma cell clone-directed therapy	0	0	0
	Lymphocytic clone-directed therapy	5	4/1/0	0
	Nonclone-directed therapy	5	2/0/3	1
Gowda *et al.*^8^	Conservative therapy	1	0/0/1	0
	Plasma cell clone-directed therapy	1	0/0/1	1
	Lymphocytic clone-directed therapy	1	0/0/1	1
	Nonclone-directed therapy	3	0/1/2	2
Gumber *et al.*^2^	Conservative therapy	2	Not mentioned^a^	2
	Plasma cell clone-directed therapy	4	0/3/1	0
	Lymphocytic clone-directed therapy	4	1/3/0	0
	Nonclone-directed therapy	0	0	0
Kousios *et al.*^10^	Conservative therapy	1	0/0/1	0
	Plasma cell clone-directed therapy	0	0	0
	Lymphocytic clone-directed therapy	3	0/3/0	0
	Nonclone-directed therapy	2	1/1/0	0
This study^b^	Conservative therapy	6	2/0/2 (N/A = 2)	2
	Plasma cell clone-directed therapy	13	4/3/5 (N/A = 1)	6
	Lymphocytic clone-directed therapy	8	1/3/2 (N/A = 2)	1
	Nonclone-directed therapy	2	2/0/0 (N/A = 0)	0

CR, complete response; ESKD, end-stage kidney disease; NR, no response; N/A: not applicable (i.e., initial proteinuria < 0.5 g/gCr); PR partial response.

Patients who reached ESKD are collected separately.

a The outcomes of the patients with conservative therapy are not mentioned.

b Kidney outcomes are based on urine protein.

Some patients even achieved CR with conservative therapy in our study and previous reports (Table 3). Considering the adverse events, it is necessary to consider which patients can be followed-up with conservative therapy. Conservative therapy may be reasonable if the amount of proteinuria is low and kidney function remains stable. Even when nephrotic range proteinuria is present, as in one of our cases, it may be possible to follow-up without clone-directed therapy if the proteinuria decreases spontaneously.

Kidney outcomes with these various therapy groups are also diverse. In a recent open-label phase 2 trial using daratumumab in 10 patients with MGRS with PGNMID pattern,^11^ of which 9 patients were without detectable clones, 4 had CR and 6 had PR in 1 year. No serious adverse events were reported. This suggests that plasma cell clone-directed therapy may be efficacious for MGRS without detectable clones. In terms of clone-directed therapy, Nasr and colleagues reported that the use of clone-directed therapy in monoclonal immunotactoid glomerulopathy was associated with better outcomes, compared to conservative therapy and steroid monotherapy, whereas 50% had positive M-protein in SPEP.^12^, ^13^ Of nonclone-directed therapy, steroid monotherapy was ineffective in our cases and in the previous reports. Zhou and colleagues reported 64 patients with PGNMID, 45 without detectable clones underwent 3 treatment regimens: steroids, immunomodulatory drugs, and bortezomib or rituximab. They showed the lowest response rate in the steroid group.^14^ There are no studies directly comparing immunosuppressive treatment or rituximab to other regimens. In our cases and in previous reports (Table 3), the heterogeneity of treatment regimens makes it challenging to evaluate the efficacy of each treatment regimen.

The extent of IFTA at diagnosis, regardless of treatment regimens, may be a key driver of kidney outcomes. We found that patients with moderate (25%–50%) to severe IFTA (>50%) had worse kidney response, consistent with the findings of a previous case series.^2^ In the study showing favorable outcomes with daratumumab,^11^ the median percentage of IFTA was as low as 12.5%. In addition, 2 patients in our cohort who achieved CR without any treatment had a low IFTA. The extent of IFTA at diagnosis may be useful in predicting treatment response and adjusting treatment intensity.

The roles of repeat kidney biopsies in MGRS are debatable. It has been reported that achieving good hematological response (i.e., very good PR or better response), was predictive of a favorable kidney outcome.^15^ Repeat kidney biopsies in AL amyloidosis were reported as not very informative, because AL amyloid deposition persists in the kidney and the kidney outcomes are driven by progression of chronic changes. In PGNMID, a case series in transplant kidney allografts reported that 14 patients (7 treated) had unchanged patterns, 7 (5 treated) had progression, and only 3 (all treated) had improvement in the repeat kidney biopsies.^16^ In another cohort of 60 patients, including 2 transplant kidney allografts, 15 underwent repeat biopsies.^4^ Nine patients showed changes in the kidney biopsies, and the most common finding was the progression from membranoproliferative glomerulonephritis to diffuse proliferative glomerulonephritis with monoclonal depositions. Although these studies did not report reasons for repeat kidney biopsies, repeat biopsies for PGNMID may be useful in some cases. First, repeat kidney biopsy may show improvement of disease activity and disappearance of depositions, so that it confirms response to treatment. Second, because IFTA are generally associated with kidney dysfunction,^17^ withholding treatment may be considered if repeat kidney biopsies show IFTA progression despite aggressive treatment. We need further research to evaluate the efficacy of repeat kidney biopsies with MGRS.

Although the identification rate of responsible clones is low among MGRS,^4^ it is possible that very small clones may exist in the bone marrow below the detection limit of currently available technologies, and more advanced technologies may be able to improve sensitivity in the future. Detection of small clones would be helpful for both treatment decisions (targeting either or both plasma cell or B cell) and following response to treatment. Serum mass spectrometry analysis of monoclonal protein may be more sensitive than SPEP with IFX. El-Khoury and colleagues evaluated the prevalence of monoclonal gammopathy in a population at high risk of multiple myeloma by SPEP, IFX, and mass spectrometry analysis.^18^ They identified the lower limit of detection for SPEP with IFX by serial dilution sensitivity testing was as low as 0.2 g/l. In contrast, the lowest limit for mass spectrometry-based screening was quantified at concentrations of 0.015 g/l. Monoclonal gammopathy was detected in 36% of their total screened cohort, and monoclonal gammopathy with concentrations below 0.2 g/dl in 26%. Most recently, another technology called RNA-based immunoglobulin repertoire sequencing has been reported.^19^ Javaugue and colleagues examined bone marrow samples obtained from 16 biopsy-proven MGRS cases (13 AL amyloidosis, 2 cryoglobulinemia, and 1 immunotactoid glomerulopathy). By sequencing immunoglobulin lambda light chain variable segment or immunoglobulin kappa light chain variable segment, they were able to identify dominant immunoglobulin clonotypes consistent with kidney allograft even in the cases where no or very low-level monoclonal proteins were detected. Although this needs to be tested in a larger cohort, these new technologies could be useful in detecting responsible clones.

Due to the very high recurrence rate (80%–90%) of MGRS postkidney transplant,^20^ it is crucial to treat the underlying condition thoroughly pretransplant. However, lack of biochemical biomarkers to ensure the treatment response makes it challenging to determine whether and when patients with MGRS without detectable clones are suitable for kidney transplantation. Multidisciplinary discussion and guideline development by kidney transplant and hematology organizations will be of paramount importance.

Our study has limitations. First, this study is a single-center case series and the number of the cases was relatively small. However, this is one of the largest series describing the clinical outcomes of MGRS without detectable clones.^2^^,^^4^^,^^8^, ^9^, ^10^ Second, treatment regimens and follow-up intervals were at the discretion of treating clinicians without standard protocol. Third, some patients in our cohort did not have thorough evaluation of responsible clones: only 4 had peripheral blood flow cytometry, and 4 did not have documented bone marrow biopsies. Thorough investigation of pathologic clones using all available techniques should be essential for proper diagnosis of MGRS without detectable clones.

In summary, our study highlighted variable diagnostic and treatment approaches for MGRS without detectable clones in the current practice. Acknowledging limitations of current technology, our field warrants advanced and sensitive methods to detect and track small clones to guide diagnosis and treatment. Multicenter,^21^ prospective studies will be needed to determine the best treatment strategies in the future.

## Disclosure

All the authors declared no competing interests.

## References

[1] Leung N., Bridoux F., Batuman V. (2019). The evaluation of monoclonal gammopathy of renal significance: a consensus report of the International Kidney and Monoclonal Gammopathy Research Group. Nat Rev Nephrol.

[2] Gumber R., Cohen J.B., Palmer M.B. (2018). A clone-directed approach may improve diagnosis and treatment of proliferative glomerulonephritis with monoclonal immunoglobulin deposits. Kidney Int.

[3] Leung N., Bridoux F., Hutchison C.A. (2012). Monoclonal gammopathy of renal significance: when MGUS is no longer undetermined or insignificant. Blood.

[4] Bhutani G., Nasr S.H., Said S.M. (2015). Hematologic characteristics of proliferative glomerulonephritides with nonorganized monoclonal immunoglobulin deposits. Mayo Clin Proc.

[5] Jaskowski T.D., Litwin C.M., Hill H.R. (2006). Detection of κ and λ light chain monoclonal proteins in human serum: automated immunoassay versus immunofixation electrophoresis. Clin Vaccin Immunol.

[6] Hutchison C.A., Plant T., Drayson M. (2008). Serum free light chain measurement aids the diagnosis of myeloma in patients with severe renal failure. BMC Nephrol.

[7] Levey A.S., Stevens L.A., Schmid C.H. (2009). A new equation to estimate glomerular filtration rate. Ann Intern Med.

[8] Gowda K.K., Nada R., Ramachandran R. (2015). Proliferative glomerulonephritis with monoclonal immunoglobulin deposition disease: the utility of routine staining with immunoglobulin light chains. Indian J Nephrol.

[9] Guiard E., Karras A., Plaisier E. (2011). Patterns of noncryoglobulinemic glomerulonephritis with monoclonal Ig deposits: correlation with IgG subclass and response to rituximab. Clin J Am Soc Nephrol.

[10] Kousios A., Duncan N., Tam F.W.K. (2019). Proliferative glomerulonephritis with monoclonal Ig deposits (PGNMID): diagnostic and treatment challenges for the nephrologist. Kidney Int.

[11] Zand L., Rajkumar S.V., Leung N., Sethi S., El Ters M., Fervenza F.C. (2021). Safety and efficacy of daratumumab in patients with proliferative GN with monoclonal immunoglobulin deposits. J Am Soc Nephrol.

[12] Hogan J.J., Vogl D.T. (2021). Untangling immunotactoid glomerulopathy in the MGRS era. Kidney Int.

[13] Nasr S.H., Kudose S.S., Said S.M. (2021). Immunotactoid glomerulopathy is a rare entity with monoclonal and polyclonal variants. Kidney Int.

[14] Zhou H., Li M., Zeng C., Chen Z., Zhang T., Cheng Z. (2022). Efficacy of immunomodulatory drugs in combination with dexamethasone in proliferative glomerulonephritis with monoclonal immunoglobulin deposits. Kidney Int Rep.

[15] Gozzetti A., Guarnieri A., Zamagni E. (2022). Monoclonal gammopathy of renal significance (MGRS): real-world data on outcomes and prognostic factors. Am J Hematol.

[16] Said S.M., Cosio F.G., Valeri A.M. (2018). Proliferative glomerulonephritis with monoclonal immunoglobulin G deposits is associated with high rate of early recurrence in the allograft. Kidney Int.

[17] Eadon M.T., Schwantes-An T.H., Phillips C.L. (2020). Kidney histopathology and prediction of kidney failure: a retrospective cohort study. Am J Kidney Dis.

[18] El-Khoury H., Lee D.J., Alberge J.B. (2022). Prevalence of monoclonal gammopathies and clinical outcomes in a high-risk US population screened by mass spectrometry: a multicentre cohort study. Lancet Haematol.

[19] Javaugue V., Pascal V., Bender S. (2022). RNA-based immunoglobulin repertoire sequencing is a new tool for the management of monoclonal gammopathy of renal (kidney) significance. Kidney Int.

[20] Leung N., Bridoux F., Nasr S.H. (2021). Monoclonal gammopathy of renal significance. N Engl J Med.

[21] Quinn G.Z., Susanibar-Adaniya S., Lau S., Kallem R.R., Hogan J. (2021). Baseline characteristics of proliferative glomerulonephritis with monoclonal immunoglobulin deposits in the international kidney registry consortium (K-REG). https://www.asn-online.org/education/kidneyweek/2021/program-abstract.aspx?controlId=3612236.

